# Glycation end-products specific auto-antibodies in Systemic Lupus Erythematosus

**DOI:** 10.6026/97320630018127

**Published:** 2022-03-31

**Authors:** Mohd Wajid Ali Khan

**Affiliations:** 1 Department of Chemistry, College of Science, University of Hail, Hail-2440, Saudi Arabia

**Keywords:** SLE, glycation, AGEs, autoantibodies, HAS

## Abstract

Systemic lupus erythematosus (SLE) is an autoimmune disease, which is highly inflammatory. Compared to a healthy control group, SLE patients exhibit a higher concentration of advanced glycation end products (AGEs) and a lower concentration of receptors for AGEs (RAGE) in serum, however, the exact aetiology is still unclear. In the present study, non-enzymatic glycation induced modification of human serum albumin (HSA) has been studied by biophysical techniques. Glycated HSA (G-HSA) was used as an antigen, and serum autoantibody levels were estimated in SLE and normal humans (NH) against it, using direct binding ELISA and competitive inhibition ELISA. Compared to N-HSA, remarkable structural modifications were observed in G-HSA. Modified HSA also showed increased pentosidine fluorescence (213.7 ± 13.4 AU). Glycation of HSA induced a conversion of α-helix and random coil to β-sheet and β-turns. Serum immuno assays results exhibited significantly (p < 0.001) higher binding of G-HSA with serum autoantibodies from SLE patients when compared with native HSA (N-HSA). Furthermore, competitive ELISA results showed significantly (p < 0.001) high percent inhibition of serum IgG from SLE patients with modified antigen. Chronic inflammation with excessive oxidative stress in SLE patients could be a possible reason for structural alterations in blood proteins, generating highly immunogenic unique new-epitopes. These in turn induce the generation of specific autoantibodies against G-HSA that may serve as a potential biomarker for SLE pathogenesis.

## Background:

Systemic lupus erythematosus (SLE) is a chronic inflammatory disease, which is also autoimmune in nature. The exact aetiology of the disease is still unknown thus, the available medicines and approved therapies are not sufficient to properly manage the disease. B cells are involved in the production of autoantibodies; hence, their depletion or inactivation is also a potential SLE treatment option [1]. A range of different factors, such as immune dysfunction of cells such as B cells, dendritic cells and neutrophils as well as genetic and environmental effects can be implicated in the pathogenesis of SLE is attributed to dysfunction of different immune cells such as B cells, dendritic cells [2]. The formation pathogenic immune complexes mediate the disease by molecular mimicry or endogenous antigen modification followed by autoantibody expression, that in turn cause tissue damage [3,4]. Non-enzymatic glycation is a condensation reaction of monosaccharides and reactive amino acid groups located on intracellular and extracellular proteins. The resultant Schiff base intermediates undergo slow Amadori rearrangement to yield stable glycated protein adducts [5]. Further irreversible chemical changes to these protein-glucose adducts may lead to the formation of advanced glycation end products (AGEs). Advanced glycation of proteins causes gluco-oxidation and renders them immunogenic [6]. Evidence that AGEs are antigenic has led scientists to hypothesize that *in vivo* AGE structure may wield a larger autoimmune response [7]. Recently autoantibodies against glycated proteins such as immunoglobulins have been detected in rheumatoid arthritis (RA), diabetes, as well as in the elderly healthy individuals [6,8-11]. Gluco-oxidative damage to proteins is increasingly being implicated in diabetes mellitus, RA, artherosclerosis, Alzheimers, amyloidosis and aging [8,9,12-14]. Albumin is the most commonly found protein in human blood and hence most susceptible to non-enzymatic glycation [15]. Thus, it is important to validate the presence of autoantibodies against G-HSA in SLE patient sera to infer the role of gluco-oxidative HSA in inducing autoimmunity in SLE.

### Chemicals and reagents:

HSA, dinitrophenylhydrazine (DNPH), Millipore filter (0.2 µm), Protein A-Agarose, Nitro bluetetrazolium (NBT), anti-human IgG conjugated to alkaline phosphatase, para-nitrophenyl phosphate, and Tween 20 were procured from Sigma-Aldrich. D-glucose was obtained from Merck. Flat bottom ELISA plates (96 wells) were bought from NUNC. Chemicals used were of analytical grade.

### Collection of blood samples:

Out of 25 SLE patients recruited for the study, 14were male and 11females [mean disease duration 7.4 ± 4.5 years; mean age 44 ± 13 Algomail-Clinic. Twenty-five (15 males and 10 females) age matched healthy volunteers (NH) with no symptoms or signs of any disease were selected. Informed consent was obtained subjects before collection of samples. The Institutional Ethics Committee, College of Medicine, UAA (Ref. No. uaa10-med113), granted ethical approval for the study. Samples were stored at -20°C. Clinical and demographic characteristics of RA patients and NH subjects are shown in Table 1(see PDF) (sex, mean age, disease duration, samples sensitive to RF, fasting blood glucose (FBG), percentage of HbA_1C_ and oxidative stress by serum carbonyl content). Fasting blood glucose levels and percent of HbA_1C_ were investigated by the central investigation laboratory using glucose oxidase and capillary electrophoresis methods, respectively.

### Modification of HSA by glucose:

HSA was glycated using our previously published procedure with slight changes [15,16]. Briefly, HSA solution (1 mg/ml in 20 m MPBS, pH = 7.4) was incubated with glucose (25 mM) for 12 weeks. Spectrophotometric determination of protein concentration was at 280nm with an absorption coefficient E^1%^_1cm_280 nm= 5.3 M ^-1^cm ^-1^ [15, 16].

### Biophysical characterization of G-HAS:

#### Tryptophan fluorescence:

An excitation wavelength of 285 nm was used to determine fluorescence intensity for tryptophan residue in the N-HSA and G-HSA. Emission was recorded between 290-440 nm [17]. Slit widths used were 10 nm. Protein solutions used were of identical concentration (100 µM). Hitachi model F2700 spectrofluorometer (Japan) was used to analyse the samples.

#### AGE-pentosidine fluorescence:

Fluorescence specific for pentosidine was applied to detect the pentosidine residue in both glycated and non-glycated samples. Excitation wavelengths of 375 nm and peaks were observed between 330–420 nm [18]. Samples used in this assay were at the concentration of 60 µM.

#### Circular dichroism (CD):

CD of both (N-HSA and G-HSA) samples (2.5µM) were recorded as published previously [15-19]. N-HSA and G-HSA results for CD analysis were expressed in milli degrees. Each sample was recoded three times and average ± SD values were calculated and given. A wavelength of between 200 - 230 CD was used to record the samples with 5 mm/milli degree sensitivity. Preparation of sample solution was in 20 mM sodium phosphate buffer, pH 7.4. Secondary structural elements were quantified based on Chen and Yang equation [19].

### Biochemical analysis of serum samples:

#### Serum carbonyl content:

Carbonyl content bound to protein in SLE (n= 25) and NH (n=25) serum samples was quantified [20]. Results for all the samples were calculated in number of nanomoles of carbonyl/mg of protein using ε379 = 22,000 M ^-1^.cm ^-1^.

#### Serum pentosidine detection by ELISA:

Competition ELISA kit was used to detect pentosidine in serum samples of individuals of all groups (FSK pentosidine ELISA kit; Fushimi Pharmaceutical, Kagawa, Japan) as described previously [11]. Briefly, pronase was added to sera samples and incubated at 55°C for 90 min. To facilitate enzyme inactivation, the mixture was heated in a water bath for 15 min. PBS containing 0.5 ml/l Tween 20 buffer was used to was samples. The pretreated serum sample and antibodies specific for pentosidine were mixed and incubated at 37°C for 60 min. Polyclonal rabbit anti-human IgG peroxidase conjugated antibodies were then added and incubated at room temperature for 60 min. After color development was arrested as per kit instructions, absorbance was measured at 450 and 630 nm, respectively. Standard curve was prepared by measuring the same standard solution of pentosidine used to quantify pentosidine in sera samples [11].

### Immuno assay of serum samples:

#### Direct binding ELISA:

ELISA was performed in polystyrene immunoplates (PolysorpTM), as described earlier [6,10&^11^]. Plates were coated with 100 µl (10µg/ml) of N-HSA or G-HSA as antigen for 2-4 hours at room temperature. TBS-T (20 mM Tris, 2.68 mM KCl, 150 mM NaCl, pH 7.4, containing 0.05% Tween-20) was used for plate washing (3-5 x). After washing theun-bound sites were blocked with 150 µl of 2 % skimmed milk in TBS (10 mM Tris, 150 mM NaCl, pH 7.4). Incubation was at room temperature for 4-6 hours. Post incubation plates were washed (3x) with TBS-T. Hundred micro liters of each TBS-T diluted serum sample was adsorbed at room temperature for 2-4 hours. TBS-T was used to wash plates (3 - 5x). Anti-human IgG alkaline phosphatase conjugate antibodies bound to antigen were assayed using p-nitrophenyl phosphate as substrate. Absorbance was recorded using automatic Accuris USA Absorbance Micro-plate Reader (MR9600-E) at 410 nm.

#### Competitive ELISA:

A previously published competitive-binding assay was used to determine antigen-antibody interaction specificity [6,10&^11^]. Hundred microliters of antigen (N-HSA or G-HSA) at a concentration of 5µg/ml were coated onto microplate's wells for 2-4 hours at room temperature. TBS-T was used to wash plates (3-5x) after incubation. Skimmed milk (2 %) was used for blocking followed by incubation for 4-6 hours at room temperature. Micro titre plates were washed (3 x) with TBS-T. In serum test tube varying concentrations (0-20 µg/ml) of inhibitors (N-HSA or G-HSA) were mixed with identical concentrations of serum IgG (10 µg/ml) for 2-4 hours at room temperature. Serum IgG without antigen served as control. This mixture was added to antigen-coated microplates. Residual antibody levels were detected at 410 nm using Accuris USA Absorbance Microplate Reader (MR9600-E).

Percent inhibition = 1 - (A _inhibited_ / A _uninhibited_) X 100

Where, A _inhibited_ is the absorbance at 20 µg/ml of inhibitor concentration and A _uninhibited_ the absorbance without inhibitor.

### Statistical evaluation:

Values are represented as arithmetic mean ± SD. Multiple comparisons were made by student t test using SPSS16 software program. Values of p< 0.05 were considered to be statistically significant.

## Results:

### Biophysical characterization of glycated HAS:

#### Tryptophan fluorescence:

Tryptophan fluorescence was quantified in both native and modified samples of HSA. The fluorescence intensity of a sole tryptophan residue located in HSA molecule was chosen to probe subtle structural and conformational changes. Analyses were conducted by using an excitation wavelength of 285 nm on samples (HSA or G-HSA). The emission maxima of native HSA (25.1 ± 3.1 AU) and G-HSA (60.9 ± 5.3 AU) samples were 330 and 320 nm, respectively. A blue shift of 10 nm for G-HSA (Table 1 - see PDF) was observed. The fluorescence intensity of G-HSA significantly (p < 0.001) increased.

#### Pentosidine specific fluorescence:

Pentosidine is a fluorescent AGE compound generated by the sequential glycation of proteins. Thus, detection of pentosidine using specific fluorescence is proof of *in vitro* glycation of HSA in our reaction samples. The optimum excitation wavelength for pentosidine (275 nm), was used on samples. A significantly high (p < 0.001) pentosidine-specific fluorescence (213.7 ± 13.4 AU) was observed for G-HSA. However, the fluorescence for native HSA (6.7 ± 0.7 AU) (Table 1 - see PDF) was insignificant.

#### Circular Dichroism: 

Glycation induced secondary structural changes in HSA (Figure 1 - see PDF) can be uncovered by CD. The CD signal of proteins in the spectral region of 200-250 nm can be attributed to secondary structure. Native HSA and modified HSA exhibited markedly different CD spectra. This is indicative of substantial secondary structural modifications in the modified HSA sample. A dip at 212.5 nm observed in the spectrum for G-HSA signifies a stable β-sheet structure. Software based on Yang equation was used to calculate secondary structural differences between native and modified HSA samples [15]. Appreciable changes were observed in HSA with respect to α-helix, β-sheet, β-turns and random coil. A decrease in α-helix and random coil structures (5.6% and 3.5% respectively) was detected in modified HSA, compared to the native form. However, β-sheet and β-turns both showed increases of 8.0% and 2.1% respectively after glycation. These findings suggest that glycation induces structural changes, accompanied by a partial destruction of the secondary structure of HSA.

### Biochemical analysis of serum samples:

#### Carbonyl contents:

Carbonyl contents present *in vivo* is a biomarker for the high levels of free radicals i.e., oxidative stress. Estimation of oxidative stress levels in SLE and NH subject samples is given in Table 2(see PDF). A significant increase in protein bound carbonyl contents (p < 0.001) in SLE subject sera as compared to NH subject sera was found. SLE patients exhibited higher quantities of (2.6 ± 0.6nmoles/mg protein) of carbonyl compound sin comparison to NH (1.4 ± 0.3 nmoles/mg protein)subjects (Table 2 - see PDF).

#### Pentosidine levels in serum samples:

Pentosidine levels were detected in each serum sample obtained from SLE and NH subjects. Increased levels of pentosidine were found in subjects with SLE (0.0267 ± 0.0021µg/ml) compared to the pentosidine levels in NH subjects (0.0248 ± 0.0025µg/ml) (Table 2 - see PDF).

#### FBG and HbA_1C_ levels in serum samples:

For each sample, FBG was estimated by glucose oxidase method, and HbA_1C_ by capillary electrophoresis method. Levels of FBG and HbA_1C_ were slightly increased in SLE samples, compared to the samples from NH subjects (Table 2 - see PDF). This increase may be attributed to inflammatory conditions in SLE patients.

### Serum immuno assays:

#### Direct binding ELISA:

Blood samples of SLE patients (n= 25) and NH subjects (n= 25) were screened for autoantibodies against G-HSA and N-HSA as antigens using direct binding ELISA. Direct binding ELISA results (Figure 2 - see PDF) showed that G-HSA was strongly recognized by SLE serum antibodies (0.593 ± 0.071) compared to healthy subjects (0.095 ± 0.029). Whereas, with N-HSA negligible binding was observed in the serum samples of both SLE (0.097 ± 0.026) and NH (0.082 ± 0.022) subjects (Figure 2 - see PDF).

#### Competition ELISA:

Specificity of serum autoantibodies was further investigated by competition ELISA. Native and modified HSA were used as competitors in the assay. Competition ELISA results showed high specificity between serum IgG from SLE and G-HSA, summarized in Table 3(see PDF). The mean maximum percent inhibition (MMPI) was found to be 54.9± 2.9 and 11.9 ±2.1 for G-HSA and N-HSA, respectively in SLE subjects. However, IgG from control subject's sera showed very little reactivity with either of the antigens (10.7 ± 2.1 and 9.6 ±2.0 for G-HSA and N-HSA, respectively).

## Discussion:

Presence of autoantibodies, such as anti-nuclear antibodies (anti-dsDNA), is well-known markers for SLE [20]. This proves the abnormal functioning of the immune system. This immune imbalance leads to chronic inflammation and hence causes other secondary complications (nephropathy, atherosclerosis, and osteoarthritis). The search for a better prognostic potential marker for SLE diagnosis that could be used in early diagnosis and disease management is ongoing. Oxidative stress is involved in the immunopathogenesis of SLE [21]. A recent study showed excessive production of ROS, along with many other factors may induce SLE [22]. Increase in cell death and delay in clearance of these dead cells, as well as apoptotic cells are commonly associated with SLE induced excessive production of ROS [23,24].There are evidences of link between oxidative stress, generation of AGEs and SLE [25-27]. Complex and unwarranted reactions may cause the formation of AGEs, which are identified by the immune system as neoepitopes [28,29]. This study was designed based on the simultaneous presence of AGE-protein and high oxidative stress which occurs in the natural state in individuals. *In vitro* glycation of HSA was carried out with glucose, and its resultant effects on HSA molecules were characterized by biophysical as says to understand the structural alterations. The conformational changes in HSA due to modifications by glycation were evaluated by tryptophan specific fluorescence. Specific changes in the microenvironment structure of HSA molecule, where tryptophan is located, were observed. Formation of AGEs in *in vitro* HSA glycation established by the detection of pentosidine is a known AGE molecule. Glycation exerts secondary structural alterations which were ascertained by a sensitive technique i.e. CD. CD helps in elucidating the changes occurring in protein on a secondary structural level. Glycation of HSA induced a conversion of α-helix and random coil to β-sheet and β-turns. SLE and NH serum samples were analysed for the presence of oxidative biomarker i.e., carbonyl content. Carbonyl content in the serum samples of SLE subjects were significantly high compared to the healthy individuals. A recent finding that showed a relation in the excessive production of ROS in SLE patients [21] also supports this result. In some studies, accumulation of AGEs was found in the skin of SLE patients [30,31]. A positive correlation was observed between AGEs accumulation and disease duration. It has been suggested that AGEs were predominantly found in tissues compared to plasma [26]. Whereas pentosidine levels were detected in fewer SLE subjects, increased levels of another AGE, 'fructosamine' were detected in all the SLE patients [32]. Another research study evaluated increased levels of pentosidine, and also found a positive correlation between SLE disease activity index and AGEs concentration [33]. Findings of this study showed that SLE samples exhibited increased levels of AGE molecule as compared to NH subjects. It is evident that the formation of AGEs occurs in SLE patients. However, the exact etiology contributing to the formation of these molecules is unknown [28,29].

Other factors such as FBG and HbA_1C_levels also differed in SLE and NH subjects. Slightly higher levels of average FBG and HbA_1C_ were detected in SLE samples as compared to the samples from NH subjects. Increased levels of FBG and HbA_1C_ might be associated with excessive inflammatory conditions in SLE patients [1-3].Glycation of blood proteins causes gluco-oxidation and may render them immunogenic [6 - check with author]. The antigenic properties of AGEs contribute to the auto immune response observed in patients with SLE [7]. Autoantibodies against AGEs have been implicated in various other autoimmune diseases such as RA and diabetes [6 - check with author, 8-10]. Thus, G-HSA was chosen as an antigen and serum autoantibodies were screened in SLE sera samples. Healthy individuals served as controls and were also screened for autoantibodies. Direct binding ELISA results from this study exhibited significantly increased levels of serum autoantibodies in SLE patients against G-HSA, whereas healthy individuals showed negligible binding. To further confirm that the autoantibodies in the SLE sera are specific to modified antigen (G-HSA), competitive ELISA assays were run for all SLE samples as well as in NH serum samples. Results showed that IgG from SLE patients exhibited strong recognition of G-HSA as compared to N-HSA. Negligible binding patterns were observed for NH serum IgG with both N-HSA and G-HSA. According to these immune assay findings and other clinical data, (carbonyl content, pentosidine, FBG and HbA_1C_) from SLE patients, is has been suggested that generation of higher amount of serum autoantibodies against G-HSA can be correlated with oxidative stress and chronic inflammation in SLE patients.

## Conclusion:

Role of G-HSA in SLE immunopathogenesis was established in this study. G-HSA has been found to be immunogenic and produce increased amounts of autoantibodies in SLE patients. It can be suggested that glycation of blood proteins might cause imbalance in humoral immunity and may further lead to the generation of antigen-antibody immune complexes, in turn contributing to the pathogenesis of SLE disease.
